# Expression of Genes and Their Polymorphism Influences the Risk of Knee Osteoarthritis

**DOI:** 10.1155/2017/3138254

**Published:** 2017-10-09

**Authors:** Abhishek Mishra, Rajeshwar Nath Srivastava, Sachin Awasthi, Devendra Parmar, Priya Mishra

**Affiliations:** ^1^Centre for Advanced Research, King George's Medical University, Lucknow, India; ^2^Department of Orthopaedic Surgery, King George's Medical University, Lucknow, India; ^3^Department of Orthopaedic Surgery, Dr. Ram Manohar Lohia Institute of Medical Sciences, Lucknow, Uttar Pradesh, India; ^4^Developmental Toxicology Division, Indian Institute of Toxicology Research, Lucknow, Uttar Pradesh, India; ^5^Department of Prosthodontics, King George's Medical University, Lucknow, India

## Abstract

**Introduction:**

Genetic factors including the level of expression of the fingerprint of genes involved in the development of bones and cartilage such as GDF-5 or ESR-*α* or CALM-1 are known to be strong determinants of the osteoarthritis (OA) in Caucasian and Oriental populations. Because of high prevalence of OA in Indian population and availability of limited genetic data, we determined whether similar genetic factors are involved in Indians as well.

**Methods:**

A case control study was carried out involving 500 patients of knee OA and equal number of healthy controls. Genotyping analyses in whole blood, mRNA, and protein expressions in peripheral blood lymphocytes (PBLs) were performed using established protocols.

**Results:**

Our results showed a significantly decreased level of mRNA and protein expressions for GDF-5, ESR-*α*, and CALM-1 genes in PBLs of OA cases when compared to healthy controls. The frequency of variant genotypes of these genes was also increased significantly in cases of OA compared to controls.

**Conclusion:**

Our results demonstrated that the decrease in expression of GDF-5, ESR-*α*, and CALM-1 in PBLs and association of polymorphism in these genes may be important in predicting the severity and thereby the progression of OA in Indian population.

## 1. Introduction

Osteoarthritis (OA), characterized by gradual loss of articular cartilage in the joint, is a leading cause of disability among the elderly people [1, 2]. Though the etiology and pathogenesis of OA is largely unknown, OA is a chronic degenerative condition of mobile joints, primarily a noninflammatory disorder characterized by an imbalance between the synthesis and degradation of articular cartilage leading to classic pathological change of wearing away and destruction of cartilage [3]. Multiple risk factors including age, obesity, tissue injuries, and gender are well established for OA [4]. It has been reported that about 80% of population has radiographic evidence of OA by the age of 65 years of which only 60% were symptomatic [5, 6]. It is estimated that more than 30–40% of Indian population over 50 years suffer from OA [7]. The prevalence and clinical severity of OA are reported to be much higher in postmenopausal women, suggesting a potential role of estrogen in the pathogenesis of OA [8].

Genetic factors such as growth differentiation factor-5 (GDF-5), a member of TGF-*β* superfamily, estrogen receptor-*α* (ESR-*α*), an important mediator of signal transduction and calmodulin-1, and a ubiquitous Ca+ binding protein which are involved in the development, maintenance, and repair of bone and cartilage appear to be associated with the etiopathogenesis of OA [8–10]. Sequencing studies have shown that SNP in GDF-5 (C/T; rs143383) influences OA susceptibility by reducing transcriptional activity leading to decrease in cartilage synthesis [11]. Previous studies have suggested an association between GDF-5 (C/T; rs143383) polymorphism and risk of knee OA in Caucasian and Oriental population [9, 11–14]. Recent study from our laboratory has also shown that polymorphism in UTR region of GDF-5 (C/T; rs143383) plays an important role in development of knee OA [13, 14]. Likewise, polymorphism in ESR-*α* is reported to be significantly associated with risk to OA development. A significant increase in the frequency of A allele (ESR-*α*, rs2228480) in patients has been associated with reduced cartilage formation that may lead to increased risk to OA [15, 16]. Functional analysis has also indicated that the variant allele (rs12885713) of CALM-1 reduces the expression of the major cartilage matrix genes (Col 2a1 and Agc 1) that enhances the susceptibility for osteoarthritis through modulation of chondrogenic activity [17]. CALM-1 gene polymorphism was reported to be significantly associated in Japanese population with OA, while no such association was observed in Han Chinese and UK population [10, 17, 18].

In contrast to Caucasians and Oriental populations, not much data is available on the association of polymorphism in GDF-5, ESR-*α*, and CALM-1 with OA in the Indian population. The present case control study was therefore initiated to investigate the association of polymorphism in GDF-5, ESR-*α*, and CALM-1 gene with OA in Indian population. As OA has been shown to be of multifactorial origin, attempts were also made to explore the association of genotype combination of ESR-*α*, CALM-1, and GDF-5 in modifying the susceptibility to OA. To study the functional role of GDF-5, ESR-*α*, and CALM-1 in OA, expression of these genes was also studied in knee OA patients [19]. As preliminary study from our laboratory has shown that GDF-5, ESR-*α*, and CALM-1 are expressed in peripheral blood lymphocytes (PBLs), attempts were also made to investigate the alterations in the expression of these genes in PBLs isolated from healthy control and knee OA cases that would help in identifying the role of expression of these genes in development of OA.

## 2. Materials and Methods

A case control study of 500 healthy controls with no sign of knee OA and 500 patients suffering of knee OA was carried out. The patients fulfilled American College of Rheumatology (ACR) clinical and radiographic criteria of OA [20]. Radiographic findings of knee OA were classified into mild (KL grade 2), moderate (KL grade 3), and severe (KL grade 4). The Average Body Mass Index (BMI) was 23.39 ± 2.39 and 25.41 ± 3.23 in controls and cases, respectively. Among the OA cases, 205 were males and 295 females ranging from 40 to 72 years with mean age of 56.15 ± 9.33 and 55.67 ± 8.58 years in males and females, respectively. In the controls, 224 were males with mean age of 54.95 ± 8.09 years and 276 females with mean age of 55.52 ± 9.04 years.

The protocol for research work was approved by the Human Ethics Committee of King George's Medical University, Lucknow. The protocol conforms to the provisions of the declaration of Helsinki in 1995. Informed consent was obtained from the study subjects for inclusion in the study. Before the collection of blood samples, it was ensured that subject anonymity was preserved. The control and cases were asked to fill out the detailed questionnaire regarding their occupation, socioeconomic status, medical history, lifestyle habits, and so on.

## 3. DNA Isolation and Genotyping Studies

Approximately 1 ml of blood was collected into citrate containing tubes from all the subjects. DNA was isolated from whole blood with the Flexi Gene DNA kit (Qiagen, CA) following the manufacturer's protocol. The method of Southam et al. 2007 [9] was followed for identifying and determining the rs143383 polymorphism in GDF-5. In brief, the PCR reaction was digested with BSiE1 restriction enzyme. Formation of two fragments (106 bp and 91 bp) and three fragments (197, 106, and 91 bp) indicated the CC and CT genotype respectively. Undigested PCR fragment was indicative of the TT genotype.

Polymorphism in ESR-*α* gene was detected by the method of Jin et al. 2004 [16]. In brief the PCR product formed was digested with BtgI for identifying (rs2228480) polymorphism. The polymorphisms in CALM-1 were detected by the established methods [10, 21]. Digestion of PCR product with ApaLI or ApeKI led to the identification of polymorphism in CALM-1 (rs12885713 and rs3814843). Third SNP of CALM-1 (rs2300496) was detected by TaqMan assay using RT-PCR. The reaction was performed using an ABI 7900 HT Fast Real time PCR system (50°C for 2 minutes, 95°C for 10 minutes, 95°C for 15 sec, and 60°C for 1 minute, for 40 cycles) and analyzed using an SDS RQ manager 1.2 software according to the manufacture's instruction.

For quality control, 10% of the samples were selected randomly and regenotyped to provide further confirmation of the results. These were found to be in 100% agreement.

### 3.1. RNA Isolation and Gene Expression Analysis by RT-PC

Total RNA was extracted from whole blood with TRI-BD (Sigma, USA) according to the manufacturer's protocol. The protocol utilizing TRI-BD reagent, a monophasic solution of phenol and guanidium isothiocyanate, is an improvement to the single-step RNA isolation developed by Chomczynski and Sacchi in 1987 [22]. The ESR-*α*, CALM-1, and GDF-5 mRNA expression were quantified using reverse transcriptase-PCR. Equal amounts of RNA were reverse transcribed using the Superscript first-strand cDNA synthesis kit with Oligo-dT (Invitrogen, USA) and diluted in nuclease-free water (Ambion) to a final concentration of 5 ng/*μ*L. Expression of housekeeping gene *β*-actin served as a control to normalize values. Targets were detected and quantified in real time using the ABI Prism 7900 sequence detector system (PE Applied Biosystems; Foster City, CA, USA) and SYBR green chemistry (Applied Biosystems, USA). Relative expression was calculated using the ΔΔCt method.

### 3.2. Isolation of Lymphocytes and Protein Expression by Western Blot

Lymphocytes were isolated from the blood by the method described previously by Dey et al. 2005 [23]. In brief after sonication peripheral blood lymphocytes (PBLs) isolated from case and control were used for immunoblotting. The blood lymphocytes were separated by sodium dodecyl sulfate–polyacrylamide gel electrophoresis (SDS–PAGE; 3% stacking gel and 7.5% separating gel) and electroblotted on Immobilon-P membrane (Millipore USA). The membranes were incubated with the primary antibodies raised against GDF-5, ESR-*α*, and CALM-1 (1 : 1000 dilution) in 5 ml of phosphate buffered saline containing 0.02% Tween-20 and 0.02% sodium azide and PBST for 3 hours at room temperature. The membranes carrying lymphocytes were incubated with 1 : 2000 dilution of rabbit anti-rabbit IgG-horseradish peroxidase (secondary antibody). Following incubation, membranes were washed 5 times with PBST (5–10 min each) and then processed for detection with chemiluminescence substrate. The membranes were visualized on VERSA DOC Imaging System (Model 1000, Bio-Rad, USA) and quantitative analysis using Quantity One Quantitation software (Bio-Rad). Prior to studying protein expression of these genes, normalization of proteins in the lymphocytes was carried out using beta-actin antibody.

### 3.3. Statistical Analysis

Genotype or allele frequencies of ESR-*α*, GDF-5, and CALM-1 among cases and controls were determined for Hardy–Weinberg equilibrium (HWE) using standard chi square statistics. The haplotype analyses (haplotype frequency estimation and pairwise linkage disequilibrium between the SNPs) were carried out using Haploview (https://www.broad.mit.edu/mpg/haploview/). Using binary logistic regression models, the relationship of ESR-*α*, CALM-1, and GDF-5 gene polymorphisms with risk of OA was determined. Combination between genotypes was also estimated by conditional logistic regression. Student's* t*-test was employed to calculate the statistical significance between control and case groups. All statistical analyses were performed with the SPSS software package (version 16.0 for windows; SPSS Chicago, IL). The power of the present test results was >80% with 95% significance level analyzed by power genetic association analysis software (https://dceg.cancer.gov/bb/tools/pga).

## 4. Results

### 4.1. Genotype Analysis

The distribution of genotypes of ESR-*α*, CALM-1, and GDF-5 is summarized in Tables 1–3. The genotype frequency of variant genotype of rs2228480 of ESR-*α* was increased in cases when compared to the controls. This increase in frequency of variant genotype was significantly associated with increased risks to knee OA. On gender-wise stratification, a significant association was observed in the females; further near to significant difference was observed in the males.

An overrepresentation of variant genotypes of CALM-1 gene was found in cases when compared to the controls. The prevalence of variant genotype of CALM-1 (rs 12885713) was increased in cases (31.60%) when compared to the controls (28.44%). Likewise the frequency of variant genotype of rs2300496 was found to increase in the cases when compared to the controls. The variant genotype of CALM-1 (rs3814843) was also found to significantly increase in cases resulting in increased risk to OA. On gender-wise stratification, this polymorphism was significantly associated in both males and females. The frequency of TT genotype of GDF-5 (rs143383) was also found to be significantly higher in cases (39.80%) when compared to the controls (26.20%). The increase in the frequency of TT genotypes in cases significantly increased the risk for OA. When the cases were stratified on the basis of gender, the frequency of TT genotype compared to CC genotype was significantly increased in cases in men (33.17%) when compared to the controls (22.32%) that significantly increased the risk to OA in male cases. In females too, the frequency of TT genotype was higher in cases (44.40%) when compared to the controls (29.34%).

Haplotype approach revealed that seven possible haplotypes of CALM-1 were observed in both cases and controls (Table 4). Haplotype of SNPs of CALM-1 gene was also found to be associated with OA susceptibility. A significant increase in the distribution of haplotype, TGC, carrying variant alleles of all three polymorphisms (T-rs12885713 or G-rs3814843 or C rs2300496) was observed in cases when compared to the controls. This increase in frequency of haplotype, TGC, resulted in increased risk to OA. When the haplotype in cases was stratified on the basis of gender, the frequency of TGC haplotype was significantly increased in cases among men that significantly increased the risk when compared to the controls. In females, the frequency of TGC haplotype was also higher in cases when compared to the controls that increased the risk to OA. This increase in risk was found to be relatively lower in females when compared to the males.

**Figure 1:**
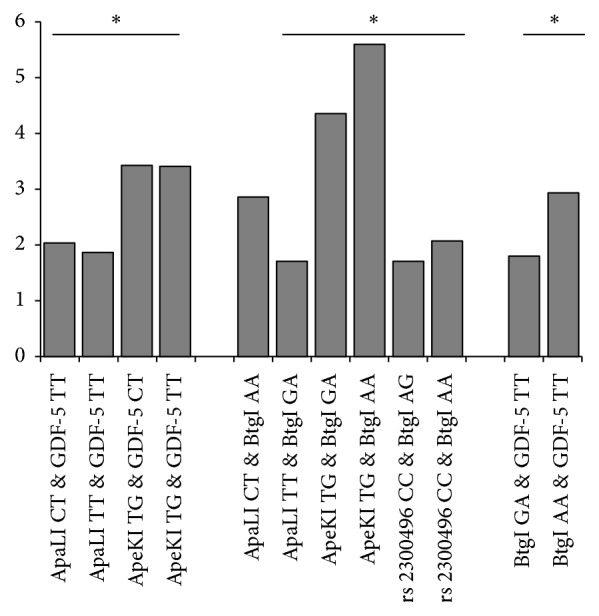
Risk associated with GDF-5, ESR-*α*, and CALM-1 genotype combinations in knee OA. Figure contains only significant OR of genotype combination of various candidate gene in knee OA. ^*∗*^*p* < 0.05 is considered statistically significant.

Differences were also observed in the distribution of combination of variant genotypes of ESR-*α*, CALM-1, and GDF-5 in cases when compared to the controls (Figure 1). The combination of variant genotype of CALM-1 (rs12885713 or rs3814843 or rs2300496) and GDF-5 (rs143383) was found to be overrepresented among cases. The combination of variant genotype of CALM-1 (rs12885713) and GDF-5 (rs143383) or CALM-1 (rs3814843) and GDF-5 (rs143383) or CALM-1 (rs2300496) and GDF-5 (rs 143383) increased the risk (2.0- to 3.5-fold) in cases when compared to the controls. Likewise, the combination of CALM-1 (rs12885713 or rs3814843 or rs2300496) and ESR-*α* (rs2228480) also increased the risk (2- to 6-fold) to OA. Significant increase in risk (1.81- to 2.93-fold) was also observed in cases carrying combination of a variant allele of ESR-*α* (rs2228480) and GDF-5 (rs143383).

### 4.2. Expression Study

**Figure 2:**
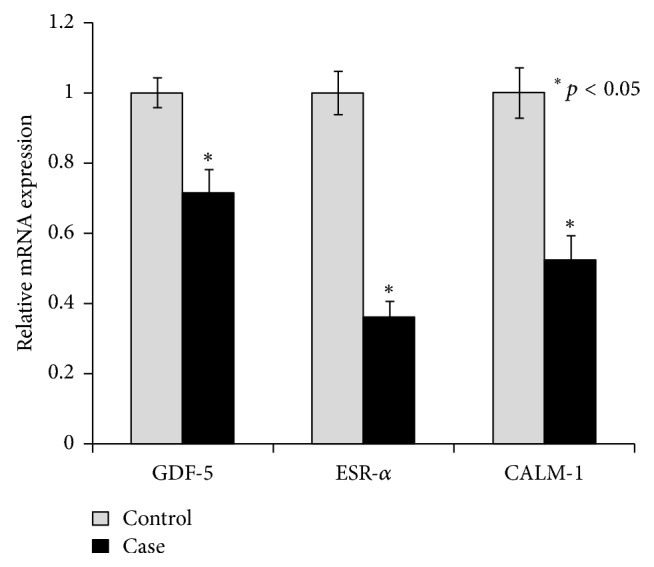
Quantitative real time PCR analysis was performed for relative mRNA expression of genes involved in cartilage synthesis and repair in control and case study. Quantitative analysis suggested that expression of GDF5, ESR-*α*, and CALM-1 genes downregulated in case group compared to their respective control. *β*-Actin served as housekeeping gene for normalization. Values are expressed as mean ± SEM (*n* = 24). ^*∗*^*p* < 0.05 versus control.

**Figure 3:**
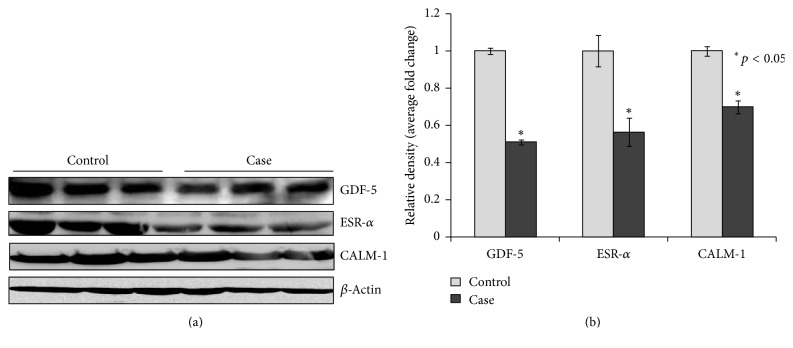
(a) Western blot analysis was performed to understand the protein level of GDF5, ESR-*α*, and CALM-1 in control and case study. (b) Bar diagram showing the relative protein density after normalization with *β*-actin. Relative protein density of GDF5, ESR-*α*, and CALM-1 was significantly decreased in case study as compared to control group. Representative blots showing three samples from each group (control and case). Values are expressed as mean ± SEM (*n* = 24). ^*∗*^*p* < 0.05 versus control.

Real time PCR was performed to examine expression at the mRNA level. Data were normalized against *β*-actin mRNA and expressed as mean ± SEM of the control group. The levels of mRNA expression of GDF-5, ESR-*α*, and CALM-1 were found to be significantly (*p* < 0.05) reduced in OA cases when compared to controls (Figure 2). We also carried out immunoblotting studies in PBLs to determine the expression of these genes at the level of protein. Our results showed similar reduction in the level of protein expression for each gene (GDF-5, ESR-*α*, and CALM-1) in protein expression as the seen mRNA expression, when compared to controls (Figures 3(a) and 3(b)).

## 5. Discussion

Based on our data from this study we report that ESR-*α*, CALM-1, and GDF-5 are polymorphic in Indian population. The frequency of variant allele of rs2228480 polymorphism of ESR-*α* gene is similar to that reported in Indian population and Caucasians but significantly less than the Oriental population [15, 24, 25]. The frequency of variant allele (T) of rs12885713 polymorphism of CALM-1 gene was similar to that reported in Caucasians [18, 26] but relatively higher than the Oriental population [10, 17]. In contrast, low frequency (2.3%) of variant allele (G) of rs3814843 polymorphism of CALM-1 was observed in our population which is similar to that reported in the Caucasians, though relatively higher frequency of this minor allele is reported in the Oriental population [21]. The frequency of minor allele (C) of the other polymorphism of CALM-1 (rs2300496) and T allele of GDF-5 was found to be similar to that observed in Oriental and Caucasians population [9, 11, 13, 17].

Our data has shown significant alterations in the distribution of variant alleles of GDF-5, ESR-*α*, and CALM-1 in OA cases when compared to the controls. Similar to the case control studies involving Caucasians and Oriental people [9, 11, 16–18, 27], an increase in the frequency of A allele (rs 2228480) was observed in OA patients when compared with the controls. Significant increase in the frequency of risk allele (T) of GDF-5 in cases of osteoarthritis is consistent with previous report demonstrating association of polymorphism in GDF-5 with OA [9, 11]. Meta-analysis has also reported significant association of GDF-5 polymorphism with osteoarthritis [12]. Likewise, as reported in case control studies with Caucasian and Oriental population [10, 17, 18], our data indicating increase in the frequency of variant alleles (T-rs 12885713 or G-rs 3814843 or C-rs2300496) of CALM-1 gene has suggested association of CALM-1 polymorphism with OA [17]. A minor increase in the T allele frequency of (rs12885713) and significant increase in variant G allele (rs3814843) in OA patients has further provided evidence for the role of CALM-1 polymorphism increasing the risk to OA in the cases.

As GDF-5, ESR-*α*, and CALM-1 involved in the cell signaling also contribute to cartilage formation and differentiation [8, 11, 17, 28, 29], it is hypothesized that polymorphism in these genes may reduce and disrupt the formation of cartilage thereby increasing the risk to OA. The present study demonstrating the reduced mRNA and protein expression of GDF-5, ESR-*α*, and CALM-1 in PBLs isolated from OA cases has provided support to the radiological analysis indicating reduced formation of cartilage as well as its disruption in the knee OA cases. Though the data was not stratified with respect to the polymorphism in these genes, it has been shown that polymorphism in the GDF-5, ESR-*α*, and CALM-1 may further decrease the activity of these genes [8, 11, 17, 28]. The decrease in the cartilage formation as evident by increase in the number of cases with KL-3 or KL-4 grading in the OA cases with the polymorphism in GDF-5, ESR-*α*, and CALM-1 has further shown that polymorphism in these genes may result in decreased cartilage volume thereby enhancing the risk to OA.

The frequency of the haplotype T-G-C (variant of all three polymorphisms of CALM-1) was overrepresented in the cases when compared with controls, resulting in 3.0-fold increase in risk of OA. Gender-wise stratification also demonstrated that the haplotype T-G-C increases the risk to OA in females and males. Similar increase in risk to OA was reported in a study involving a Japanese population [17]. Further evidence that the risk to OA may be increased in cases carrying more than one SNP was provided by our studies indicating a much greater risk in cases carrying genotype combination of ESR-*α* (rs 2228480) and GDF-5 (rs143383) or CALM-1 (rs12885713, rs3814843, and rs2300496) and ESR-*α* (rs2228480) or CALM-1 (rs12885713, rs3814843, and rs2300496) and GDF-5 (rs143383) [17].

Interestingly, individuals with less severe knee OA (KL-2 grade) showed the lesser magnitude of the decrease in the expression of GDF-5, CALM-1, and ESR-*α* in peripheral blood, when compared to the patients with KL-4 grade of knee OA. Thus further validation of blood GDF-5, CALM-1, and ESR-*α* expression with the radiological profile (KL grade) of knee OA will help in developing peripheral blood expression profiles of candidate genes as possible biomarkers to monitor the onset and progression of OA.

In conclusion, the study demonstrates that polymorphism in genes involved in the development, maintenance, and repair of bone and cartilage such as GDF-5, CALM-1, and ESR-*α* modifies the susceptibility to OA. RT-PCR and immunochemical studies demonstrating downregulation in the expression of GDF-5, CALM-1, and ESR-*α* in the PBLs isolated from OA patients have suggested involvement of these genes in process of formation of disrupted cartilage in OA patients. Relatively higher risk alteration in cases carrying haplotype of variant alleles of CALM-1 gene and in cases carrying the combination of variant genotypes of GDF-5 and ESR-*α* (rs2228480) or CALM-1 and ESR-*α* (rs2228480) or CALM-1 and GDF-5 have demonstrated the importance of genotype combinations and the role of haplotypes in predicting the risk to OA.

## Figures and Tables

**Table 1:** Genotype association between SNP in ESR-*α* gene and knee osteoarthritis.

	All subjects			Women			Men		
	Control (%)	Case (%)	OR, (95% CI), *p* value	Control (%) 276	Case (%) 295	OR, (95% CI), *p* value	Control (%) 224	Case (%) 205	OR, (95% CI), *p* value
Genotype									
BtgI (rs 2228480)									
GG	231 (46.20)	170 (34.00)	1.00, (Ref)	127 (46.01)	94 (31.86)	1.00, (Ref)	104 (46.24)	76 (37.07)	1.00, (Ref)
GA	218 (43.60)	259 (51.8)	1.61, (1.23–2.11), 0.000^*∗*^	120 (43.47)	158 (53.55)	1.77, (1.24–2.54), 0.001^*∗*^	98 (43.75)	101 (49.26)	1.41, (0.94–2.11), 0.096
AA	51 (10.20)	71 (14.2)	1.89, (1.25–2.85), 0.002^*∗*^	29 (10.50)	43 (14.57)	2.00, (1.16–3.44), 0.011^*∗*^	22 (09.82)	28 (13.65)	1.74 (0.92–3.27), 0.083

Allele									
BtgI (rs 2228480)									
G	680 (68.0)	599 (59.9)	1.00, (Ref)	374 (67.75)	346 (58.64)	1.00, (Ref)	306 (68.30)	253 (61.70)	1.00, (Ref)
A	320 (32.0)	401 (40.1)	1.42, (1.18–1.70), 0.000^*∗*^	178 (32.24)	244 (41.35)	1.48, (1.16–1.88), 0.001^*∗*^	142 (31.69)	157 (38.29)	1.33, (1.00–1.77), 0.042^*∗*^

OR, odds ratio; 95% CI, 95% confidence interval; Ref, reference category; ^*∗*^*p* < 0.05 is considered statistically significant.

**Table 2:** Genotype association between 3 SNPs in CALM-1 gene and knee osteoarthritis.

	All subjects			Women			Men		
	Control (%) 500	Case (%) 500	OR, (95% CI), *p* value	Control (%) 276	Case (%) 295	OR, (95% CI), *p* value	Control (%) 224	Case (%) 205	OR, (95% CI), *p* value
Genotype									
ApaLI (rs 12885713)									
CC	125 (25)	102 (20.4)	1.00, (Ref)	70 (25.36)	62 (21.01)	1.00, (Ref)	55 (24.55)	40 (19.51)	1.00, (Ref)
CT	233 (46.6)	240 (48)	1.26, (0.91–1.73), 0.150	119 (43.11)	117 (39.66)	1.11, (0.72–1.70), 0.631	114 (50.89)	123 (60.00)	1.48, (0.91–2.39), 0.106
TT	142 (28.4)	158 (31.6)	1.36, (0.96–1.92), 0.078	87 (31.5)	116 (42.02)	1.50, (0.96–2.33), 0.068	55 (24.55)	42 (20.48)	1.05, (0.59–1.86), 0.867

Allele									
rs 12885713									
C	483 (48.3)	444 (44.4)	1.00, (Ref)	259 (46.92)	241 (40.84)	1.00, (Ref)	224 (50.00)	203 (49.51)	1.00, (Ref)
T	517 (51.7)	556 (55.6)	1.17, (0.98–1.39), 0.080	293 (53.07)	349 (59.15)	1.28, (1.01–1.61), 0.038^*∗*^	224 (50.00)	207 (50.48)	1.02, (0.78–1.33), 0.886

Genotype									
ApeKI (rs 3814843)									
TT	477 (95.4)	444 (88.8)	1.00, (Ref)	259 (93.8)	258 (87.45)	1.00, (Ref)	218 (97.32)	186 (90.73)	1.00, (Ref)
TG	23 (4.6)	56 (11.2)	2.61, (1.58–4.32), 0.000^*∗*^	17 (6.1)	37 (12.55)	2.18, (1.2–3.98), 0.009^*∗*^	06 (2.67)	19 (9.26)	3.71, (1.45–9.48), 0.003^*∗*^
GG	—	—		—	—		—	—	

Allele									
rs 3814843									
T	977 (97.7)	944 (94.4)	1.00, (Ref)	535 (96.92)	553 (93.72)	1.00, (Ref)	442 (98.66)	391 (95.36)	1.00, (Ref)
G	23 (2.3)	56 (5.6)	2.52, (1.53–4.12), 0.000^*∗*^	17 (03.07)	37 (06.27)	2.10, (1.17–3.78), 0.011^*∗*^	06 (01.33)	19 (04.63)	3.46, (1.39–8.57), 0.004^*∗*^

Genotype									
rs 2300496									
AA	167 (33.4)	147 (29.4)	1.00, (Ref)	86 (31.15)	80 (27.11)	1.00, (Ref)	81 (36.16)	67 (32.68)	1.00, (Ref)
AC	220 (44)	221 (44.2)	1.14, (0.85–1.52), 0.371	125 (45.28)	132 (44.74)	1.13, (0.76–1.67), 0.524	95 (42.41)	89 (43.41)	1.13, (0.73–1.74), 0.573
CC	113 (22.6)	132 (26.4)	1.32, (0.94–1.85), 0.097	65 (23.55)	83 (28.13)	1.37, (0.88–2.14), 0.162	48 (21.42)	49 (23.90)	1.23, (0.73–2.06), 0.421

Allele									
rs 2300496									
A	554 (55.4)	515 (51.5)	1.00, (Ref)	297 (53.84)	292 (49.49)	1.00, (Ref)	257 (57.36)	223 (56.82)	1.00, (Ref)
C	446 (44.6)	485 (48.5)	1.17, (0.98–1.39), 0.080	255 (46.19)	298 (50.50)	1.18, (0.95–1.5), 0.145	191 (42.63)	187 (45.60)	1.12, (0.86–1.47), 0.380

OR, odds ratio; 95% CI, 95% confidence interval; Ref, reference category; ^*∗*^*p* < 0.05 is considered statistically significant.

**Table 3:** Genotype association between SNP in GDF-5 (BsiE1) gene and knee osteoarthritis (KOA).

	All subjects			Women			Men		
	Control (%)	Case (%)	OR, (95% CI), *p* value	Control (%) 276	Case (%) 295	OR, (95% CI), *p* value	Control (%) 224	Case (%) 205	OR, (95% CI), *p* value
Genotype									
BsiE1 (rs 143383)									
CC	97 (19.40)	75 (15.00)	1.00, (Ref)	58 (21.01)	47 (15.93)	1.00, (Ref)	39 (17.41)	28 (13.65)	1.00, (Ref)
CT	272 (54.4)	226 (45.20)	1.07, (0.75–1.52), 0.686	137 (49.63)	117 (39.66)	1.05, (0.66–1.66), 0.821	135 (60.26)	109 (53.17)	1.12, (0.65–1.94), 0.673
TT	131 (26.2)	199 (39.80)	1.96, (1.35–2.85), 0.000^*∗*^	81 (29.34)	131 (44.40)	1.99, (1.24–3.20), 0.004^*∗*^	50 (22.32)	68 (33.17)	1.89, (1.03–3.47), 0.038^*∗*^

Allele									
C	466 (46.6)	376 (37.6)	1.00, (Ref)	253 (45.83)	211 (35.76)	1.00, (Ref)	213 (47.54)	165 (40.24)	1.00, (Ref)
T	534 (53.4)	624 (62.4)	1.44, (1.21–1.73), 0.000^*∗*^	299 (54.16)	379 (64.23)	1.52, (1.19–1.92), 0.000^*∗*^	235 (52.45)	245 (59.75)	1.34, (1.02–1.76), 0.031^*∗*^

OR, odds ratio; 95% CI, 95% confidence interval; Ref, reference category; ^*∗*^*p* < 0.05 is considered statistically significant.

**Table 4:** Distribution of CALM-1 haplotypes among controls (*n* = 500) and knee OA cases (*n* = 500).

Allele	Control (%)	Case (%)	OR, (95% CI), *p* value	Women			Men		
				Control (%)	Case (%)	OR, (95% CI), *p* value	Control (%)	Case (%)	OR, (95% CI), *p* value
CTA	341 (34.1)	320 (32.0)	1.00, (Ref)	167 (30.24)	166 (28.13)	1.00, (Ref)	175 (39.06)	154 (37.56)	1.00, (Ref)
TTC	290 (29.0)	315 (31.5)	1.15, (0.92–1.44), 0.193	155 (28.07)	196 (33.22)	1.27, (0.94–1.71), 0.116	136 (30.35)	119 (29.02)	0.99, (0.71–1.38), 1.000
TTA	208 (20.8)	189 (18.9)	0.96, (0.75–1.24), 0.799	125 (22.64)	119 (20.16)	0.95, (0.68–1.33), 0.797	82 (18.30)	69 (16.82)	0.95, (0.65–1.40), 0.820
CTC	138 (13.8)	120 (12.0)	0.92, (0.69–1.23), 0.604	89 (16.12)	71 (12.03)	0.80, (0.55–1.17), 0.254	49 (10.93)	49 (11.95)	1.13, (0.72–1.78), 0.578
TGC	17 (1.70)	48 (4.8)	3.00, (1.69–5.34), 0.000^*∗*^	11 (1.99)	29 (4.91)	2.65, (1.28–5.48), 0.006^*∗*^	6 (1.33)	19 (4.63)	3.59, (1.40–9.24), 0.004^*∗*^
TGA	2 (0.02)	4 (0.04)	2.13, (0.38–11.71), 0.373	2 (0.36)	4 (0.06)	2.01, (0.36–11.13), 0.414			
CGA	3 (0.03)	2 (0.02)	0.71, (0.11–4.27), 0.707	3 (0.54)	2 (0.03)	0.67, (0.11–4.06), 0.661			
CGC	1 (0.01)	2 (0.02)	2.13, (0.19–23.61), 0.527	1 (0.18)	2 (0.03)	2.01, (0.18–22.40), 0.561			

OR, odds ratio; 95% CI, 95% confidence interval; Ref, reference category; ^*∗*^*p* < 0.05 is considered statistically significant.
